# Analyzing early childhood allergy prevention motivation of mothers of infants and its predictors using latent class analysis and structural equation modelling

**DOI:** 10.1186/s12889-024-20436-6

**Published:** 2024-10-24

**Authors:** Markus Antonius Wirtz, Anja Alexandra Schulz, Andrea Heiberger, Carolin Dresch

**Affiliations:** Research Methods in Health Sciences, Faculty for Mathematics, Natural Sciences and Technology, University of Education Freiburg, Kunzenweg 21, 79117 Freiburg, Germany

**Keywords:** Parental prevention motivation, Early childhood allergy prevention, Latent class analysis, Socio-cognitive models, Structural equation modelling

## Abstract

**Background:**

Allergic diseases are among the most common chronic diseases in childhood. Early childhood allergy prevention (ECAP) behaviors of those caring for the infant during pregnancy and the first months of life may influence the risk of allergy development over the life course. Motivation and intention to use appropriate primary ECAP measures are thus of critical importance.

**Aims:**

To characterize parental ECAP motivation, (a) valid indicators will be developed and (b) typical parental characteristics will be identified. (c) According to socio-cognitive models, the predictive value of parental risk perception, control belief and self-efficacy for parental ECAP motivation shall be determined.

**Method:**

A sample of *N* = 343 (expectant) mothers of infants completed a questionnaire on self-reported ECAP motivation, risk perception, control belief, and self-efficacy. The cross-sectional data were analyzed using latent class analysis and structural equation modelling including nominal regression models.

**Results:**

Four typical maternal response profiles (motivated to a customary degree, 70%; motivated to use primary preventive measures, 17.8%; reluctant towards new prevention measures, 6.4%; highly motivated to apply preventive measures in case of an existing allergy, 5.8%) could be identified for the items on ECAP motivation. After splitting the model variables “risk perception” (allergy vs. allergy-associated general health problems) and “self-efficacy” (trust vs. insecurity) a satisfactory model-fit was achieved (CFI = .939; RMSEA = .064). Particularly, increased “risk perception-allergy” (OR = 1.655) and “self-efficacy-insecurity” (OR = 2.013) as well as lower “risk perception-general health” (OR = 0.555) and “control belief” (OR = 0.217), respectively, are associated with higher ECAP motivation.

**Conclusion:**

The use of ECAP-measures by parents to protect their newborns from allergies is important, but there are deficiencies in their implementation. Based on a social cognitive model approach, predictive characteristics could be identified, which are associated with increased motivation to implement ECAP-measures. For public health our findings provide a promising basis for conception of behavioral and environmental ECAP prevention measures and their motivated implementation by parents.

**Supplementary Information:**

The online version contains supplementary material available at 10.1186/s12889-024-20436-6.

## Introduction

Disease prevention is of particular importance in modern societies, where chronic diseases increasingly dominate the disease spectrum [1–3]. Allergic diseases, e.g., are among the most common chronic diseases in childhood [4] and may cause serious health burden over the course of the entire life [5]. Thus, effective preventive measures fostering early childhood allergy prevention (*ECAP*) should be considered as early as possible. During pregnancy and in the first months of a child’s life, there is a “window of opportunity”, i.e. a time period in which the risk of developing an allergic disease can be reduced [6–8]. Approved recommendations for primary *ECAP* are available (national guideline in Germany [9, 10]). To put effective *ECAP* recommendations into practice, parents have to be prepared and willing to integrate and adapt evidence-based recommendations into their daily child-care routines. Especially during pregnancy and in early childhood (expectant) parents are accountable for making health-related decisions for their child to ensure their best possible health development (e.g. breastfeeding [11, 12]; nutrition [13, 14]). Accordingly, proactive counseling of (expectant) mothers and the young families is a key element of modern disease prevention and health promotion strategies [15].

### The relevance of parental motivation in the field of primary prevention

In public health prevention *motivation* has to be regarded as a central determinant of health behavior, as implementing of health promotion and prevention measures requires more than just being informed and health-conscious [3, 16–21]. Determinants of behavioral decisions and action regulation (i.e. mental planning, controlling and adjusting activities to achieve specific goals) must be specifically taken into account both in the health education of individuals and in the design of public health communication (e.g., obesity prevention [22, 23], dental health [24], body dissatisfaction or eating disorder prevention [25]).

Hence, parental *motivation* and behavioral *intentions* are of crucial importance for the application of preventive measures [26–28]. The theoretical construct *motivation* describes cognitive processes that involve setting and appraising goals [29]. *Motivation* results from motives and can be understood as the willingness to act. It is assumed that *motivation* to act is determined by the desirability of the goal sought in each case and by its perceived feasibility [30, 31]. Accordingly, parents can be regarded as motivated if they consider the reduction of their child’s allergy risk important, and preventive measures to be effective and implementable [32].

Motivation and health behavior models [30, 33, 34] emphasize that persons may generally be convinced of the usefulness and benefits of preventive behavior, but not necessarily willing or determined to put it into practice. This is why models of health behavior underline the importance of *volition*. *Volition* is present when people plan the concrete implementation of specific behaviors or actions in order to achieve defined goals. *Intention formation* must therefore be taken into account as a prerequisite for concrete action: The clearer and more concrete people’s *intention*, the more likely the behavior will be executed. But even the presence of an *intention* does not suffice for the action to be carried out for certain. Because actions only correspond to *intentions* with some restrictions, the so-called *intention-behavior gap* and methods for bridging this gap are a particular focus of health education research.This intention-behavior gap is known to be distinctly pronounced for primary preventive behaviors in parents [35]. To support parental action in line with parental *intentions*, it is beneficial if the prevention behavior to be implemented is concretely defined, the implementation context is clearly specified and the behavior is to be carried out rather promptly [36]. Kruglanski, Chernikova and Rosenzweig [37] emphasize the importance of *motivational readiness*, which denotes the inclination to act in accordance with a desire, regardless of whether this action is ultimately realized or not. Thus, *motivational readiness* concerns being prepared for a future action, *Motivation* provides a drive to perform an action, and *Intention* represents the deliberate decision to perform a particular action. *Motivational readiness* and *motivation* to perform *ECAP* interventions are the focus of this study, which asks women about attitudes and behaviors that are relevant indicators of the individual’s disposition for *ECAP* behaviors in childcare.

To characterize different types of *motivation*, it has proven appropriate to distinguish different profiles, which reflect typical patterns of motivational aspects in which people differ systematically. Schwarzer and Fleig [38], for example, identified health behavior profiles that characterize unmotivated, motivated and acting individuals. Identifying different groups of people regarding their motivation to engage in health-conscious behavior, was found to be a valid modeling approach in various health-related areas (nutrition [39]; physical activity [40]; alcohol consumption [41]). Davis, Coleman and Kramer [42] identified typical maternal profiles regarding breastfeeding that relate to the extent of infant-centered caring (see also: [11]). In particular, intervention recommendations must be tailored to the individual motivational constitution. It is therefore advisable to first find out the personal characteristics of the mothers so that support can be tailored to their individual personality (i.e. profile- or person-centered approach [43, 44]) .

### Determinants of parental prevention motivation oriented on social cognitive process models

*Parental prevention motivation* needs to be understood and defined in the context of the preceding characteristics that may influence it, and its action-related consequences (i.e., reduction of the child’s health risks) [30]. According to established social cognitive models (e.g. *HAPA*-*model* [34, 45–47]; *Theory of planned behavior* [25, 33]) *risk perception* (i.e., belief that the child is at risk), *self-efficacy* (i.e., belief in one’s ability to execute actions successfully), and *outcome expectancy* (i.e., belief that the behavior leads to targeted effects) are crucial for the individual to be prepared and willing to engage in a particular preventive behavior [28, 48]. With regard to *parental prevention motivation*, *risk perception* is based on the subjective assessment of parents’ perceived vulnerability of their child’s health as well as the severity of the according health threat: “*Could potential development of allergy threaten the child’s health in the medium or long term?*” [49]. *Risk perception* initiates a process of thinking and examining the possible consequences of preventive actions [50]. *Self-efficacy expectancy* describes parents’ subjective belief to be able to accomplish health preventive tasks based on their own competencies [34, 51]. It is a crucial determinant for goal setting, effort investment, persistence, and relapse probability [45]: “*Do parents feel able to influence the infant’s health development through their own behavior?*”. Jones and Prinz [52] confirmed that parental *self-efficacy expectancy* is an important characteristic that can predict parenting success as well as risks to child development. Thus, it should be considered a starting point for prevention interventions.

The latter determinant for *intention formation* are *outcome expectations*, including weighing the pros and cons of a particular preventive behavior [34]: “*Do parents belief that their behavior effectively results in a reduction in the child’s allergy risk?*” Especially in the case of long-term prevention of health risks, *outcome expectations* are closely related to *control beliefs*. *Control beliefs* refer to parents’ beliefs about their capability to control the child’s allergy risk through their own actions [53]. *Health-related control beliefs* are assumed to be composed of *contingency beliefs* (e.g., “*My behavior will reduce the risk of allergy in the long term*”) and *efficacy beliefs* (e.g., “*I am able to reliably implement this behavior*”). According to this structural distinction, the *outcome expectations* formulated in the HAPA model correspond essentially to the subcomponent *contingency beliefs* [54, 55].

### Research goals

This study focuses on the relationships between the model-based determinants of parental *ECAP motivation* and typical profiles of parental *ECAP motivation*. These may contribute to a better understanding of effective individual-centered as well as environmental public health *ECAP* interventions. If parents are subjectively convinced that they are in control and self-efficient with their established behavior, they could be less likely to change behavioral routines (*self-confident steady*). On the other hand, parents assuming to have higher control and *self-efficacy* may also be more confident and positive about alternative *ECAP motivation* behaviors that are considered useful (*self-confident ready to improve*). If parents have a low sense of control and perceive themselves as having limited *self-efficacy* regarding the child’s allergy development, this could on the one hand be associated with being more willing to improve their behavior in the sense of applying new *ECAP* recommendations (*insecure willing to improve*). On the other hand, they may be more critical of behavioral changes due to their own uncertainty of being able to influence the child’s susceptibility to allergies and lack of expectation of success (*insecure change-skeptical*).

To gain a better understanding of mothers’ *motivation* to actually implement *ECAP* measures in their caring behavior, this study pursues the following objectives.

Research goal 1: To identify typical profiles characterizing *ECAP motivation* of (expectant) mothers of infants.

Research goal 2: To identify a theory-based model representing the determinants *risk perception*, *control belief* and *self-efficacy expectancy* of *ECAP motivation*.

Research goal 3: To determine the predictive power of the model based predictors for *ECAP motivation* profiles. It is assumed, that.

- *risk perception*, *control belief* and *self-efficacy* are direct predictors of *ECAP motivation* profiles (direct predictive effects), and.
- the effect of *risk perception* on *ECAP motivation* profiles is additionally mediated by *control belief* and *self-efficacy* (partial mediated predictive effects). The effect of *control belief* on *ECAP motivation* profiles is additionally mediated by *self-efficacy* (partial mediated predictive effects).

## Materials and methods

### Data collection

The German-wide cross-sectional survey took place between May 2021 to March 2022. The main inclusion criteria for participation were either being pregnant or parenting of a child up to a maximum age of 3 years. In addition to the (expectant) mothers, their partners could also participate. Due to the low participation rate of the partners (39,9%), this study is limited to the (expectant) mothers. This is also beneficial to prevent biases, because the information provided by partners cannot be regarded as independent of each other. This would lead to biased statistical estimates, because statistical assumption would be violated (i.e., assumption of stochastic independence: the information given by one person must not be influenced by another person in the sample) [56]. The sample was recruited German-wide adopting the snowball method within social networks in particular [57]. Furthermore, gatekeepers (e.g., day care centers, midwives, pediatricians) in all 16 federal states of Germany were contacted by e-mail and telephone, and a cooperation with a health insurance company was established. An expense allowance of 30€ was paid for full participation. Informed consent was obtained for all participants, and all participants were informed about the study objective and the protection of personal data.

For data collection three-part online questionnaire was used (processing time approx. 45 min each, approx. 10 days apart) in German language. Sociodemographic information and all psychological characteristics (e.g., *self-efficacy expectancy*) were collected in part 1 of the questionnaire. Part 2 and 3 focus on the assessment of health literacy in the areas of COVID-19 and allergy prevention and are reported elsewhere [58–60]. The item contents were translated into English for this paper. The program Tivian (Tivian XI GmbH) was used for the online survey.

### Survey instruments

Sociodemographic data (e.g., age, education, socioeconomic status) were recorded at the beginning of the survey and follows the demographic standard of the German Health Interview and Examination Survey for Adults [61]. The MacArthur scale was used to assess subjective perceived socioeconomic status [62].

*Parental primary prevention motivation on Allergy*:To assess indicators of *ECAP motivation* 10 items were defined. Item formulation was oriented on the *Health Regulatory Focus Scale* (*HRFS*; [63–65]). *Health Regulatory Focus* is defined generically as the “individual’s tendency to use promotion or prevention strategies in the pursuit of health goals” ( [64], p. 452) and is thus directly suitable for assessing *motivation* and *motivational readiness* as described in the introduction. The translation of the HRFS items is attached in Appendix A. The wording of the generated items translated into English is also shown in Table 1.

**Table 1:** Study sample characteristics (N = 343)

	M (SD) / [Min, Median, Max] or n (%)
Age	32.15 (4.72) / [18, 32, 50]
**Women with at least one child** Number of children Age of youngest child in month Pregnant women	281 (82%) 1.48 (0.85) / [0, 1, 5] 15.6 (10.94) / [0, 13, 50] 62 (18%)
Primipara	170 (52.19)
Subjective socioeconomic state (MacArthur scale)	5.87 (1.42) / [1, 6, 10]
**Marital status**	
Married Single, but living with a steady partner Single parent Registered civil partnership Other	240 (70%) 87 (25%) 14 (4%) 1 (0.3%) 2 (0.6%)
Nationality: German	331 (97%)
Mother tongue: German	329 (96%)
**School graduation**	
University entrance qualification / High school diploma (“Abitur“) Advanced technical college entrance qualification Secondary school diploma (“Mittlere Reife“) Elementary school diploma (“Hauptschulabschluss“) Other	156 (45%) 27 (8%) 139 (41%) 15 (4%) 6 (2%)
**Occupation**	
Employee Civil servant Worker Vocational training / qualification Self-employed University graduate in liberal professions Other	227 (66%) 26 (8%) 24 (7%) 15 (4%) 6 (2%) 7 (2%) 3 (1%)

*Parental Risk perception* was assessed by using three different kinds of questions. First, two items assess the subjective probability that (a) allergies will be an important issue in the child’s life in the long term and (b) the child’s quality of life will be significantly impaired by allergic diseases (6-point Likert scale). A second part assesses the risk of the child to develop an allergy compared to the risk of other children. The response is given on a seven-point scale from “1” = “much lower risk than other children” to “7” = “much higher risk than other children”. Thus, the recommendation of Schwarzer et al. [34] was followed to assess *risk perception* aspects also by estimating the probability of the according disease. A third group of questions contains nine items that assess the subjectively perceived threat posed by allergies (e. g. “I fear that my child’s health development will be significantly affected by allergies”). The statements were rated using a six-point scale ranging from “1” = “does not apply at all” to “6” = “applies to a very high degree”.

*Parental Control Belief*: The items of the instrument *Control Beliefs about Illness and Health* [66]) served as the basis for the development of the item pool for the survey of parental *control belief*. The original version comprises 21 items (six-point Likert scale: “1” = “not true at all” to “6” = “very true”), which are assigned to three dimensions of *internal*,* external*, and *fatalistic control beliefs* (7 items each). Its subscales show a satisfactory reliability (*Cronbach’s ﻿α * = .64 − .77) [66–68]. Due to the limited data collection time within the comprehensive HELICAP assessment, 9 items (3 items of each facet) were selected for the survey in the present study. Since the three subscales are substantially correlated (indicating a strong common source of variance *control belief*) and the formation of a cross-facetted generalized self-concept scale is also established for the original instrument [66], the expression of the cross-facetted overall construct *control belief* was thus to be measured.

*Parental self-efficacy expectancy*: The operationalization was based on Jerusalem and Schwarzer’s *Scale for General Self-Efficacy Expectation* [69], which is a reliable assessment (Cronbach’s α = .71-.89; Retest reliability (1-year interval): r_tt_ = .54). Item formulations had to be adapted because mothers’ *self-efficacy* expectations regarding child’s health (external health prevention), rather than her own health, should be assessed. In total, the scale contains 10 items, answered on a six-point Likert scale (“1” = “completely disagree” to “6” = “completely agree”).

To ensure content validity and usability, the items of all scales adapted for the topic *ECAP* were tested using cognitive interviews with *n* = 14 parents [70]. The final assessment is attached in Appendix B.

### Data analysis

For descriptive statistics, item and scale analysis SPSS 29 was used. Mplus 8.8 was used to conduct latent class analysis (LCA; research goal 1), confirmatory factor analysis (CFA; research goal 2) and structural equation modelling (SEM; research goal 3) [71]. As all questions had been marked as mandatory questions in the online survey, there were no missing values in the data set.

To identify different profiles of *ECAP motivation*, LCA was used. LCA assumes that typical response profiles (latent classes/latent categorical variables) determine the entire data information. The analysis approach of the LCA determines (1) which number of *ECAP motivation* profiles can be assumed as underlying, (2) which proportion of women belong to each *ECAP motivation* profiles, and (3) by which response profile women of *ECAP motivation* profile i are characterized [72]. LCA is a likelihood-based procedure that identifies (1) probabilities of women’s responses and (2) the probabilities of each woman belonging to a particular latent *ECAP motivation* profile i [73]. Model estimation is conducted by using maximum likelihood estimation (MLR). Model fit has been compared for 1 to 10 classes based on the Bayesian information criterion (BIC). The lower the BIC value, the better the model fit. One-way analysis of variance (ANOVA) was used to test for *ECAP motivation* item differences between the identified *ECAP motivation* profiles. For the interpretation of the effect size eta-squared (η^2^) following thresholds are used: .01 = small, .06 = medium, .14 = strong [74].

To analyze the predictive characteristics for *ECAP motivation* profiles a two-stage SEM procedure was adopted following Kline’s recommendations [56]. First, CFA of the underlying characteristics (i.e. factors) was estimated (research goal 2). The basic assumption of CFA is that any item is a distinct indicator of only one underlying latent characteristic. Different latent characteristics are assumed to be associated, i.e. intercorrelated [56]. To ensure that each item is sufficiently associated with the underlying characteristic, the item information should be determined to at least 40% (i.e. indicator reliability ≥ .4; factor loading ≥ .63; [75]) by the according underlying characteristic (factor). Factor reliability of each underlying characteristic should exceed the critical value 0.6. To ensure sufficient factor discrimination, each factor should be associated more closely with its own items than with other factors (square root of average variance extracted > factor intercorrelations; [76]). In a second step, SEM was calculated to answer the research question 3 regarding the relationships between the analyzed underlying characteristics and their predictive value for *ECAP motivation* profile membership. SEM has been conducted utilizing the restricted maximum likelihood algorithm (MLR) for categorical data [77]. A good model fit to the data is indicated if the comparative fit index (CFI) and the Tucker–Lewis index (TLI) achieve values close to or above 0.95, the standardized root mean square of the residuals (SRMR) is less than 0.05, and the root mean error of approximation (RMSEA) value is .08 or less [56, 78]. RMSEA and SRMR are measure of the amount of data information, that remains unexplained by the model.

## Results

### Sample characteristics

A total of 343 (expectant) mothers of infants, including *n* = 62 pregnant women (18%, mean week of gestation: 24.8 (*SD* = 9,8; range: 3–40) and *n* = 281 mothers with children aged 0–3 years (82%) were included in the study. Self-reported socioeconomic status is slightly higher in the sample (*M* = 5.87; *SD* = 1.42; *median* = 6) than in the German reference standard sample of women aged 18–44 years (*M* = 5.45; *SD* = 1.50; [62]. The mean age of the respondents was 32.2 years (*range*: 18–50; *SD* = 4.7; median: 32). The majority (*n* = 193, 56%) were affected by at least one allergy, while most children (*n* = 291, 85%) did not suffer from any allergy so far. Further characteristics of the sample can be found in Table 2. Since the proportion of women with university entrance qualifications (54%) corresponds to that in the general population, the educational status can be considered to be appropriately distributed in the sample.

**Table 2:** ECAP motivation item characteristics for overall sample and for the identified ECAP motivation profiles as well as test values of ANOVA between profile groups

ECAP motivation Items^1)^	Sample (*N* = 343)	ECAP motivation				ANOVA (Profiles)		
		Profile 1 *N* = 240 (70%)	Profile 2 *N* = 61 (17.8%)	Profile 3 *N* = 22 (6.4%)	Profile 4 *N* = 20 (5.8%)			
	M^4)^ (SD)	M(SD)	M(SD)	M(SD)	M(SD)	F	*p*	η^2^
ECAPM09: It is important to me to do everything that could help to protect my child’s health.	5.45 (0.75)	5.42 (0.73)	5.49 (0.85)	5.36 (0.79)	5.80 (0.52)	1.76	.161	(.015)
ECAPM08: If my child’s health was harmed by a wrong or thoughtless decision of mine, I could never forgive myself.	4.99 (1.06)	4.93 (1.05)	5.02 (1.10)	5.14 (1.04)	5.35 (1.09)	1.15	.332	(.010)
ECAPM06: It’s easy for me to try new things if they might benefit my child’s health.	4.55 (1.06)	4.49 (1.04)	4.77 (1.02)	4.23 (1.07)	5.05 (1.15)	3.42	.018	.029
ECAPM07: I’m thinking about what I can do to prevent an allergy for my child.	4.44 (1.27)	4.34 (1.24)	4.85 (1.18)	3.86 (1.42)	5.10 (1.17)	6.26	< .001	.053
ECAPM03: I do everything I can to implement the mentioned recommendation as good as possible.^2)^	4.28 (0.97)	4.15 (0.80)	4.79 (0.90)	2.95 (0.84)	5.75 (0.44)	52.30	< .001	.316
*ECAPM05: I [do not] prefer traditional and established methods to new recommendations.* ^*2,3)*^	3.92 (1.02)	3.81 (0.93)	4.74 (0.87)	3.23 (0.87)	3.55 (1.28)	21.33	< .001	.159
*ECAPM04: […] general recommended measures are helpful for me and my child.* ^*2,3)*^	3.71 (1.16)	3.64 (0.89)	4.89 (0.80)	1.86 (0.71)	3.00 (1.59)	67.34	< .001	.372
ECAPM10: For my child’s health, the topic of allergies has a particularly high importance.	3.70 (1.15)	3.57 (1.01)	4.10 (1.08)	3.18 (1.18)	4.55 (1.40)	9.15	< .001	.075
*ECAPM01: I [would also] follow the mentioned recommendation [although] my child [doesn’t have] an allergy.* ^*2,3)*^	3.04 (1.24)	2.87 (0.92)	4.67 (0.89)	1.77 (0.75)	1.40 (0.50)	108.70	< .001	.491
*ECAPM02: I [would also] follow the mentioned recommendation if health risks [could not] be certainly excluded.* ^*2,3)*^	2.37 (1.08)	2.39 (0.92)	3.13 (1.25)	1.27 (0.46)	1.00 (0.00)	37.41	< .001	.253

^(1)^ Original wording, see Appendix B. Sorted in descending order according to mean values in the total sample. ^(2)^ Relates to a fictitious predefined recommendation for effectively reducing a child’s allergy risk; ^3)^*Italics*: reformulated inverted items; the information in square brackets do not correspond to the original wording. ^4)^ The higher the mean values the more likely people agree with the statement (“1” = “do not agree at all” to “6” = “do agree completely”)

### ECAP motivation profiles identified by LCA

Table 2 depicts the 10 *ECAP motivation* items in descending order according to average ratings. The mean values are generally in the upper range of the scale (3.70 corresponding to “rather disagree” to 5.45 corresponding to “agree”). The highest levels of agreement are reported for the statements that it is important to do everything possible to protect the child’s health (ECAPM09) and that parents could not forgive themselves if their child’s health would be harmed by their careless decisions (ECAPM08). Lower agreement appears only for the use of measures when no allergy is present (ECAPM01; M = 3.04) and risks cannot be excluded with certainty (EAPM02; M = 2.37).

The LCA revealed that a 4-profile solution (*BIC* = 5077.39; 3-profile solution: *BIC* = 5077.70, 5-profile solution: *BIC* = 5081.59) accounts best for the data information. Except for the two items with the highest general agreement ECAPM08 and ECAPM09, the *ECAP motivation* items differs significantly between the four identified profiles. The one-factor ANOVA shows significant (*p* < .018 / .001; Table 2) differences between the identified profile groups for the other eight items. For five items, the effect size η^2^ indicates a statistically large effect (> 0.14). Table 2 shows the profile-specific parameters. For the identified *ECAP motivation* profiles, the following typical characteristics emerge:

*Profile 1 – Motivated in a customary degree* (*n* = 240; 70%): For this group, the values of the analysis items are similar to those in the overall sample, i.e., when no distinction was made according to typical profiles (i.e., latent classes).

*Profile 2 - Motivated to use primary preventive measures* (*n* = 61; 17.8%): Women characterized by this profile indicate a comparatively high readiness to use preventive measures, even if the child does not suffer from allergies (ECAPM01) and risks associated with the measures cannot be ruled out with certainty (ECAPM02). The individuality of the child (ECAPM04) and traditional methods (ECAPM05) are considered to be of secondary importance in the decision to use preventive measures.

*Profile 3 – Reluctant towards new prevention measures* (*n* = 22; 6.4%): These women are significantly more skeptical about the use of new prevention measures (ECAPM06). The issue of allergy is of minor importance (ECAPM07, ECAPM10) and preventive measures would only be considered if the child would suffer from allergy (ECAPM01). The tendency to use traditional and established methods is comparatively high (ECAPM05) and for new measures risks should be certainly excluded (ECAPM02). The individuality of the child is considered very important (ECAPM04).

*Profile 4 - Highly motivated to apply preventive measures in case of an existing allergy* (*n* = 20; 5.8%): Women characterized by this profile report a highly pronounced willingness to implement prevention measures (ECAPM03, ECAPM06, ECAPM07). They consider the topic of allergies and their avoidance to be central (ECAPM10). They are particularly concerned that the child’s health could be harmed by a wrong or careless maternal decision (ECAPM09). Doubts about the implementation of general recommendations (ECAPM04) and the preference for traditional methods in contrast to new recommendations (ECAPM05) are of minor importance for women belonging to this profile group. In contrast to these ratings, however, these women proved to be unexpectedly reserved regarding primary preventive (ECAPM01) use and if risks cannot be ruled out with certainty (ECAPM02).

### Factorial analysis of items indicating maternal risk perception, control belief and self-efficacy

To analyze items representing *risk perception*,* control belief* and *self-efficacy*, a CFA model was specified in which all items were defined as indicators of the according factors. However, the measures of the global model fit (*TLI* = .674; *CFI *= .698; *RMSEA* = .085; Table 3) indicated these models did not adequately fit the data. To identify misspecification in the postulated measurement models, we considered (i) the strength of item-factor associations (criterion for sufficient association: *indicator reliability* > .4) and (ii) critical residual correlations (*r* > .25 [56]), .

**Table 3:** Measures of global fit for the structural equation models

	x²	df	*p*	x²/df	TLI	CFI	RMSEA
Threshold for acceptable fit			> 0.05	< 3	≥ .90	≥ .90	≤ .080
Original-Model (3-factorial)	1498.998	431	< 0.001	3.478	.674	.698	.085
Modified Model (5-factorial)	222.184	93	< 0.001	2.389	.921	.939	.064

In the *risk perception* domain, it proved necessary to split the item group. Six items (Table 4) reflect the main factor *risk perception – allergy* adequately (factor reliability = 0.90). The second factor *allergy-associated general health problems* (*AAGHP*; *factor reliability* = .69) is indicated by the two items assessing the risk that allergies are associated with physical and psychological health problems (RP05, RP06). From the original 12 items four items (risk relative to other children, thoughts about allergy risk, increasing societal relevance of allergies, fear of allergies) were eliminated due to insufficient indicator reliability. Thus, in the final model, *risk perception* is represented by the two moderately correlated factors *risk perception - allergy* and *AAGHP*: Mothers who reported an increased risk of allergic disease in their child were also more likely to see allergic disease as associated with psychological and physical sequelae (*r* = .325).

**Table 4:** Measures of local fit and relevant item properties (*N* = 343).

Allergy-associated general health problems (AAGHP; 1 = Does not apply at all; 6 = Applies to a very high degree)						
	**M**	**SD**	**Skewness**	**IR**	**C.R.** ^1)^	FR: .69 AVE: .53 α: .68
RP05: Association allergies and psychological problems.	3.46	1.227	-0.178	.375	8.189	
RP06: Association allergies and physical problems.	4.10	1.062	-0.692	0.729	9.068	
**Risk perception – Allergy** (1 = Does not apply at all [^*^very unlikely]; 6 = Applies to a very high degree [* very likely])						FR: .90 AVE: .62 α: .88
RP01: Probability: Long-term relevance of allergies in the life of the child.^*,3)^	3.91	1.432	-0.238	.392	11.831	
RP02: Probability: Impairment of the child’s quality of life due to allergies.^*,3)^	3.42	1.290	0.084	.469	14.786	
RP07: Concern about the child having or getting allegies.	3.83	1.265	-0.138	.602	31.058	
RP08: Importance of the subject of allergies.	3.83	1.326	-0.191	.563	27.386	
RP09: Concern that child’s quality of life is affected by allergies.	3.66	1.326	-0.014	.857	61.233	
RP12: Concern that the child’s health development will be affected by allergies.	2.99	1.085	0.376	.430	19.542	
**Self-efficacy – Insecurity** (1 = Completely disagree; 6 = completely agree)						FR: .80 AVE: .66 α: .79
SE02: Uncertainty because something could be made wrong.	3.18	1.211	0.448	.767	18.448	
SE04: Feeling of being overstrained.	2.73	1.121	0.700	.862	20.605	
**Self-efficacy – Trust** (1 = Completely disagree; 6 = completely agree)						FR: .78 AVE: .48 α: .77
SE03: Making the right decisions	4.37	0.645	-0.143	.588	15.293	
SE05: Find solution in case of problems	4.55	0.690	0.111	.399	15.727	
SE07: Simply to act as I think is right	4.47	0.822	-0.415	.457	17.525	
SE08: Trust in own judgment and abilities	4.55	0.743	-0.100	.632	24.378	
**Control Belief** (1 = not true at all; 6 = very true)						FR = .67; AVE: .51 α: .67
CB-SH-01^2)^ (Items CB01, CB03, CB05, CB07, CB09)	2.75	0.534	0.164	.646	5.899	
CB-SH-02^2)^(Items CB02, CB04, CB06, CB08)	2.86	0.652	0.116	.413	5.717	

^(1)^*p* < .001 for all values; ^(2)^ Split-half subscores; ^(3)^ In order to take the different response format into account, these two items were modeled as indicators of an additional first-order factor

In the indicator set assumed to indicate *self-efficacy*, four items were found to be insufficiently associated with the assumed factor (behavioral (in-) security, orientation on own experiences as a child, orientation on other parents, behavioral security). In contrast to the unidimensionality reported in the literature [76], two intercorrelated (*r* = − .578) subfactors had to be specified. Accordingly, the two items SE02 and SE04 represent women’s *self-efficacy-related insecurity* (*factor reliability* = .80), whereas the four items SE03, SE05, SE07, and SE08 reflect *self-efficacy-related trust* (*factor reliability* = .78). In the original model, the required value for satisfactory *indicator reliability* of .4 was not achieved by 8 of the 9 items measuring *control belief*. Thus, the information of the underlying factor is insufficiently reflected in the individual items. Nevertheless, the reliable estimation of *control belief* could be achieved (*factor reliability* = .672) by aggregating the items into two parallel subscores (split-half method; [79]).

The resulting modified 5-factorial overall model exhibits satisfactory global (Table 3; e.g., *CFI* = .939, *RMSEA* = .064) and local data fit. The five predictive factors are measured reliably (*factor reliability* > .69) and are well separated (max. absolute correlation = .578 < lowest root of *AVE* = .692 [76]; Table 5).

**Table 5:** Latent intercorrelations of the factors (off-diagonal) and square root AVE (diagonal, italics) (*N* = 343)

	SE-Trust	SE-Insecurity	Control Belief	RP-allergy	AAGHP
SE-Trust	.692***	− .578***	− .132	− .084	.132
SE-Insecurity		.812***	.253***	.192*	.049
Control Belief			.714***	.143*	− .055
RP – Allergy				.782***	.325***
AAGHP					.728***

*** p <  .001; * p < .05. SE = self-efficacy; RP = risk perception; AAGHP = Allergy associated general health problems

### Modelling the predictive value of risk perception, control belief and self-efficacy for ECAP motivation profiles

**Fig. 1:**
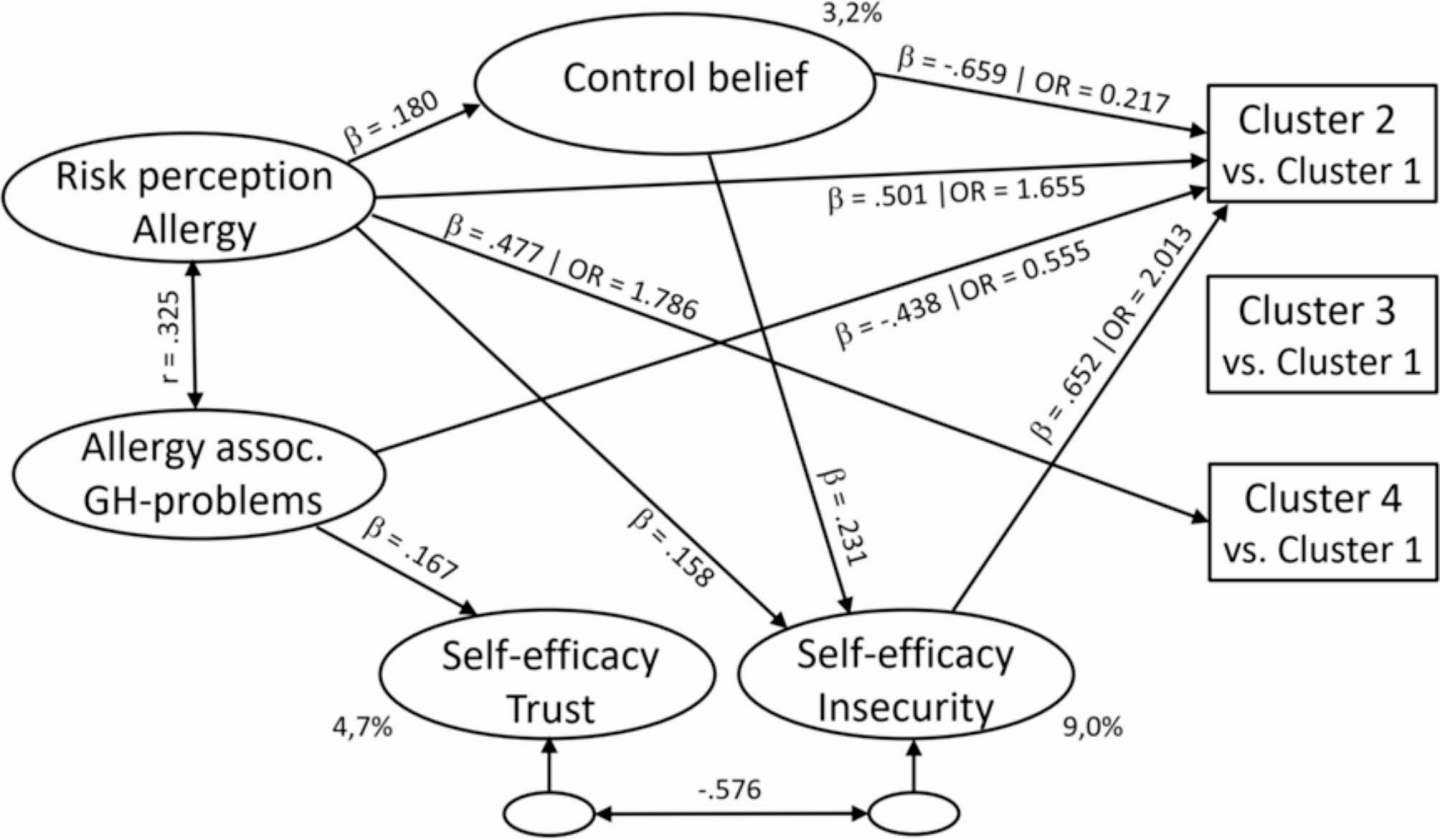
Predictive structure for the ECAP motivation profiles

Finally, the predictive value of the factors (*risk perception – allergy*,* allergy-associated general health problems*,* control belief*, *self efficacy-trust*,* self-efficacy insecurity*) for the *ECAP motivation* profiles were estimated in a structural model. Based on the HAPA model, it was further specified that *Risk perception - allergy* and *Allergy-associated* are defined as predictors of *control belief*,* self efficacy-trust* and *self-efficacy insecurity*. In addition, it is assumed that *control belief* mediates the effects of *risk perception* on *self efficacy-trust* and *self-efficacy insecurity*. The predictive value of all five factors for the *ECAP motivation* profile group membership are determined: i.e. the membership in profile group 2 (*Motivated to use primary preventive measures*), 3 (*Reluctant towards new prevention measures*) and 4 (*Highly motivated to apply prevention measures*) in reference to the largest profile group 1 (*Motivated in a customary degree*). Figure 1 shows the assumed model structure, with the prediction pathes which proved to be significant.

The probability of belonging to profile group 2 “*Motivated to use primary preventive measures*” is systematically enhanced with (1) increasing *risk perception for allergy* (*β*﻿ = 0.501), (2) decreasing *allergy-associated general health problems* (*β* = − .438), (3) decreasing *control beliefs* (*β* = − .659), and (4) increasing *self-efficacy insecurity* (*β* = .652). The predictive value of *risk perception-allergy* is also mediated by *control belief* and *self-efficacy-insecurity*. As expected, high *allergy-related risk perception* and low *control belief* result in increased *self-efficacy insecurity*, which corresponds to an increased tendency to use primary preventive *ECAP* measures.

Belonging to profile group 3 (*Reluctant towards new prevention measures*) proved to be independent of the predictors. Mothers with enhanced *risk perception of allergy* are more likely to exhibit profile 4 (*Highly motivated to apply prevention measures*).

In addition to the *β*-weights for predicting profile group membership, the odds ratios (*OR*) are listed in the figure: These indicate the factor by which the odds for the respective profile group change if the value on the predictor construct increases by one standard deviation. Values of *OR* > 1 indicate an increase in odds with increasing predictor values, *OR* values less than 1 indicate a decrease in odds with increasing predictor values.

## Discussion

In a cross-sectional survey of *N* = 343 (expectant) mothers, characteristics of primary *ECAP motivation* and its theory-based predictors *risk perception*, *control-belief* and *self-efficacy* were assessed and analysed. *ECAP motivation* of the women can be described by 4 characteristic profiles. The higher the allergy-related *risk perception*, the lower the *control-belief* and the higher the *self-efficacy*, the more likely the women were to show a favourable *ECAP motivation* profile for primary prevention.

The results on the 10 *ECAP motivation* items point to fundamental problems in the view on the relevance and benefits of primary *ECAP* measures. Women most strongly agree to the statement that it is important for (expectant) mothers to do everything that could help to protect the child’s health (ECAPM09; *M* = 5.45). This contrasts strikingly with the considerably lower level of agreement with the statement referring to the use of primary *ECAP* measures which are proven to be effective (ECAPM01; *M* = 3.04). Note that this implies a logical incongruence: The application of a proven effective primary preventive measure would have to take place in any case, if everything is done that is beneficial to the infant’s health. Furthermore, it is inconsistent that mothers report that it is important to do everything possible to implement a recommended measure although they tend to do so to a much lesser extent if the child is not allergic (*SRM*(ECAPM03 vs. ECAPM01) = 0.82). This implies that *ECAP* measures are considered as not or only limited conducive for child’s health. This discrepancy could result from the fact that mothers assume that the allergy problem will not be relevant for the child (low *risk perception*), or that the measures may not be considered effective in the individual case. Furthermore, the implementation of the measures might be considered too demanding, as parents have to be aware of multiple health prevention topics (e.g. vaccination, infection prevention, obesity prevention, sun protection; [14, 35, 80, 81]). By examining the subgroup-specific results in the latent classes representing *ECAP motivation* profiles, these inconsistencies can be understood in a more differentiated way.

### Implications of typical ECAP motivation profiles

The *ECAP motivation* profile 1 (Motivated in a customary degree; 70,0%) is very similar to the average response pattern in the overall sample. In contrast, women with profile 2 are willing to engage in the use of primary *ECAP* measures, i.e. regardless of whether the child is allergic. Thus, this rather small group (17.8%) has the desirable *motivational readiness* to engage in primary *ECAP*. It is not the presence of allergy symptoms that triggers the *motivation* to implement measures (ECAPM01), but the awareness that long-term health development should be promoted. They are also open-minded to implement new recommendations, since they have fewer reservations about new recommendations (ECAPM04, ECAPM02) and are less oriented towards traditional behavior (ECAPM05). Accordingly, mothers with profile 2 exhibit a more salutogenetic attitude [82] and seem predisposed to take advantage of the *ECAP* “window of opportunity” in pregnancy in the first months of the infant’s life [7, 8].

Mothers characterized by profiles 3 and 4 very clearly state that *ECAP* measures would not be used if the child is not allergic (ECAPM01). Hence, primary preventive measures are refused by these mothers, although they report to be willing to do everything to protect the child’s health (ECAPM09). This indicates that disease-driven preventive behavior is characteristic for these women [82]. This inconsistency is especially distinct for profile 4: a very high willingness to implement new recommendations (ECAP06), a pronounced thinking about possibilities of allergy prevention are reported (ECAP07) and a high adherence in the implementation of recommended measures is indicated (ECAPM03) [16, 83]. Mothers with profile 3 exhibit a more consistent attitude toward the rejection of primary prevention measures.

### Determinants of ECAP motivation profiles

The integrated modeling of the socio-cognitive predictors and the *ECAP motivation* profiles (Fig. 1) suggests that the willingness of mothers to apply *ECAP* measures (profile 2) seems to be determined by their particular focus on allergies. This could be due to an increased risk of allergies in the child [84]. Alternatively, these parents could assess the allergy risk more appropriately. Sicherer et al. [85], for example, have shown that parents underestimate the child’s allergy risk systematically. If the latter is true, this would provide an important approach to reinforcing parental *ECAP* behavior: If underestimating the risk of allergies could be reduced through better information and explanation, this would promote desirable parental awareness and behaviour [86]. In addition, it would be important to consider possible barriers and facilitators [35, 87] for guideline-compliant behavior. Barriers and facilitators may significantly influence maternal *motivation* and *motivational readiness*. Furthermore, they might moderate or mediate preceding intentional processes determining *ECAP* behavior [34, 46].

### Limitations of the study

We analyzed survey data, which may be affected by response sets such as social desirability, self-serving bias, or halo effects. These biasing effects may occur especially when mothers feel insecure about their own behavior and are guided by the possible judgemental reactions of others [88]. For statements on potential causal effects, it must be taken into account that cross-sectional data were analyzed. Interpretations of sequences and effects are thus to be understood as theoretical inferences and not as proven causal effects. The aim of the study was to examine maternal characteristics regardless of whether the child has an increased risk of allergies (primary preventive orientation). Although 85% of the parents stated that their child had no allergies, it could nevertheless not be ruled out that parents with a specific interest in allergy prevention were over-represented. Although our intial intention was to examine the data of couples of parents, the number of partners of the interviewed women who agreed to participate was not sufficient to conduct the analysis at the level of couples. It is important to evaluate the complementary views of fathers in the future, because child rearing and child care is jointly realized by parents. Data-based modifications were made in the modeling of the predictor characterstics using CFA. Since the aim of the present study was not to validate the item groups by scale analysis, but to obtain error-variance-adjusted estimates of the latent factors, a satisfactory CFA solution could thus be found. Nevertheless, it would have been desirable to cross-validate the adequacy of the data-based modifications in a separate sample. Because the sample sizes of *ECAP motivation* profile groups 3 and 4 are small, *n* = 22 and 20, respectively, the statistical power in estimating class membership is considerably lower than for *ECAP motivation* profile 2 (*n* = 61). This may have contributed to the prediction paths for profile group 3 and 4 membership not proving significant (increased b-error risk).

### Implications for future research and conclusions

Concerning the issue of how parents can be supported to more validly assess the nature, benefits and importance of *ECAP* measures [89] proactive counseling [82, 90] and health literacy interventions [17] should be considered. Parents should be supported in basic literacy skills enabling them to understand and utilise the impact of *ECAP* interventions (functional health literacy). Furthermore, advanced skills that enable them to interpret and tailor this information to individual life situations should be regarded (interactive and critical health literacy; [91–93]). Radzyminski and Callister [14] summarize, that infant nutritional benefits, maternal benefits, knowledge about infant feeding, and personal and professional support are the major determinants of maternal decisions on infant feeding. In addition to parental health literacy and knowledge, personal parental experiences and beliefs have to be regarded. Thus, information offers should be developed and adapted as closely as possible to the parents’ living environment [17]. Timm et al. [21] emphasize that strategies to improve parental health behaviors should involve the family environment to be effective. In particular, knowledge should be conveyed by means of role models. Furthermore, home-based activities could be established to change the home environment, and flexible communication services (e.g., digital information services or coaching contact person) could be provided to strengthen solution-oriented social support. In addition, parental beliefs (behavioral, normative, control), which are basal for *Motivation* according to the *Theory of planned behavior* [33], should be taken into account. Furthermore, the needs of parents have to be met by adequately designing the structures in the care system in which parents are involved before and after birth [18–20, 83]. Future research should strive to understand natural parent-child interactions, general health promotion and primary preventive action as integrative and complementary.

Prochaska and Prochaska [3] call for establishing more comprehensive *Health Behavior Change Interventions* for primary prevention in the context of chronic diseases. *ECAP* should also focus on ensuring that parental prevention behaviors are geared toward the holistic development of the child’s health and the strengthening of the child’s resilience. Since *ECAP* measures correspond strongly with measures that generally suport the infant’s health development (e.g. breastfeeding, hygiene behavior), *ECAP* should rather be seen as an accentuation of generally recommended preventive measures [6]. It seems likely that it is more effective to communicate the topic of *ECAP* to parents less as an additional prevention aspect that they must consider. Rather, it seems to be important to emphasize that *ECAP* and general health promotion are largely complementary. It would be interesting for future research to clarify the extent to which this framing can counteract parents’ feelings of being overwhelmed in health prevention or *ECAP* and strengthen their *control belief* and *self-efficacy* in promoting their child’s health care [21, 94].

In this study, we succeeded in developing a useful operationalization of *ECAP motivation* that allows the identification of typical profiles associated with the targeted use of evidence-based *ECAP* measures. It would be interesting to investigate whether the low tendency to use *ECAP* measures is also related to the lower public visibility of the allergy topic and the changing, possibly contradictory prevention recommendations (shifting evidence; [95, 96]). Thus, a comparison with other early childhood prevention topics would be particularly informative.

## Electronic supplementary material

Below is the link to the electronic supplementary material.

Supplementary Material 1: Appendix A: Description of the adaptation of the HRFS items for ECAP motivation

Supplementary Material 2: Appendix B: Questionnaire assessing ECAP motivation, risk perception, control belief and self-efficacy

## Data Availability

The datasets created and analyzed as part of the current study are not available publicly, but can be obtained from the authors (anja.schulz@ph-freiburg.de) upon reasonable request. The data are not made freely available to the public, as this was not explicitly requested in the ethics application.

## Abbreviations

AAGHP: Allergy Associated General Health Problems
ANOVA: Analysis of Variance
AVE: Average Variance Extracted
BIC: Bayesian Information Criterion
C. R.: Critical Ratio
CB: Control Belief
CFA: Confirmatory Factor Analysis
CFI: Comparative Fit Index
COVID-19: Corona Virus Infection Prevention 19
df: Degrees of Freedom
ECAP: Early Childhood Allergy Prevention
FR: Factor Reliability
HAPA: Health Action Process Approach
HELICAP: Health Literacy in Early Childhood Allergy Prevention
HRFS: Health Regulation Focus Scale
IR: Indicator Reliability
LCA: Latent Class Analysis
MLR: Restricted Maximum Likelihood
OR: Odds Ratio
PPM: Parental Prevention Motivation
RMSEA: Root Mean Square Error of Approximation
RP: Risk Perception
SE: Self-Efficacy
SEM: Structural Equation Modeling
SH: Split-Half
SRM: Standardized Response Mean
TLI: Tucker-Lewis Index
