# Supplementary material for: Analyzing early childhood allergy prevention motivation of mothers of infants and its predictors using latent class analysis and structural equation modelling
Source: BMC Public Health. 2024 Oct 24;24:2950. doi: 10.1186/s12889-024-20436-6 (PMC11515318; doi:10.1186/s12889-024-20436-6)
Supplement: Supplementary file 1 — Supplementary Material 1: Appendix A: Description of the adaptation of the HRFS items for ECAP motivation [file 12889_2024_20436_MOESM1_ESM.pdf]

Appendix A - Description of the adaptation of the HRFS items for ECAP motivation

The adaptation of the individual item formulations of the HRFS for the ECAP content area was item-specific and content-related. First, 5 of the 8 items of the *HRFS* were used as the basis for formulating the items on *ECAP motivation* due to their significance for *ECAP* as well. To capture motivation-related individual engagement, the original item "I frequently think about the health problem I may have in the future" (HRFS2) was included by the two items „I'm thinking about what I can do to prevent an allergy for my child" (ECAPM07) and „For my child's health, the topic of allergies is of particularly high importance" (ECAPM10). Women's openness for using new preventive measures was assessed by the item „It's easy for me to try new things if they might benefit my child's health" (ECAPM06). This wording is based on the content of the two items „I do not hesitate to embrace new experiences if I think they can improve my health" (HRFS1) and „If I see a good opportunity to improve my health, I take adavantage of it right away" (HRFS8). Since for parents the orientation on traditional methods in caring for the infant may have a significant impact the openness to new methods, the item „I prefer traditional and established methods to new recommendations" (ECAPM05) was supplemented. Parents' need to avoid own failures in caring for their child is reflected by „If my child's health was harmed by a wrong or thoughtless decision of mine, I could never forgive myself" (original HRFS7: „I often worry about mistakes I could make concerning my health"). Furthermore, sceptics about general recommendations ("Since each child is individual, general recommended measures are not very helpful for me and my child"; ECAPM04) and the fear of potential risks of new prevention measures ("I only follow the mentioned recommendation if health risks can be certainly excluded"; ECAPM02) were included [27].

The individual relevance of health in the care of the child (ECAPM09: „It is important to me to do everything that could help to protect my child's health") was also formulated oriented to

the HRFS original item content „I see myself as someone who does my utmost to improve my health“ (HRFS6). Since the present study focuses on the *motivation* of parents to implement primary preventive measures, the item ECAPM01 "I only follow the mentioned recommendation if my child indeed has an allergy" was included. Item ECAPM01 is thus intended to differentiate it from the general health protection motivation of parents recorded by item ECAPM09.

In order to formulate the items for the (expectant) parents as concretely as possible for realistic decisional situations, a situation description in the form of a vignette was formulated for items ECAPM01, 02, 04 and 05: A recommended *ECAP* measure was characterized as effectively reducing the risk of allergy or allergy symptoms. However, a change in parents' everyday behavior would be necessary to implement the recommended measure.

Moreover, some items were formulated with regard to the general health of the child when ECAP measures can be understood as a subset of general early childhood health prevention measures. Preventive action such as "exclusively breastfeeding the child for the first four to six months of life" implies generic health prevention behavior. Breastfeeding has a positive effect on the child's allergy risk but is also associated with several other health benefits for mother and child [68].

All items were formulated as statements and response were given on a six-point Likert scale from 1 = do not agree at all to 6 = do agree completely.
