# Supplementary material for: Analyzing early childhood allergy prevention motivation of mothers of infants and its predictors using latent class analysis and structural equation modelling
Source: BMC Public Health. 2024 Oct 24;24:2950. doi: 10.1186/s12889-024-20436-6 (PMC11515318; doi:10.1186/s12889-024-20436-6)
Supplement: Supplementary file 2 — Supplementary Material 2: Appendix B: Questionnaire assessing ECAP motivation, risk perception, control belief and self-efficacy [file 12889_2024_20436_MOESM2_ESM.pdf]

Appendix B - Questionnaire assessing ECAP motivation, risk perception, control belief and self-efficacy

Original item (German version)	Response categories (German version)	Translation[1)]	Response categories
**ECAP motivation**			
**ECAPM01**: Ich befolge diese Empfehlung nur, wenn mein Kind tatsächlich eine Allergie hat.[2)]	Six-point Likert scale:<br>1= Trifft überhaupt nicht zu;<br>6= Trifft in sehr hohem Maße zu	I only follow this recommendation if my child actually has an allergy.	Six-point Likert scale:<br>1 = do not agree at all;<br>6 = do agree completely
**ECAPM02**: Ich befolge diese Empfehlung nur, wenn gesundheitliche Folgerisiken sicher ausgeschlossen werden können. [2)]		I only follow this recommendation if health risks can be safely excluded	
**ECAPM03**: Ich tue alles dafür, diese Empfehlung so gut wie möglich umzusetzen. [2)]		I do everything to implement this recommendation as good as possible	
**ECAPM04**: Da jedes Kind individuell ist, sind allgemein empfohlene Maßnahmen für mich und mein Kind wenig hilfreich. [2)]		Because every child is individual, general recommended measures are not very helpful for me and my child.	
**ECAPM05**: Ich ziehe traditionelle und bewährte Methoden neuen Empfehlungen vor.[2)]		I prefer traditional and proven methods to new recommendations	
**ECAPM06**: Es fällt mir leicht, Neues auszuprobieren, wenn es der Gesundheit meines Kindes helfen könnte.		It's easy for me to try new things if they might benefit my child's health.	
**ECAPM07**: Ich mache mir Gedanken, was ich tun kann, um eine Allergie bei meinem Kind zu vermeiden.		I'm worried about what I can do to prevent an allergy in my child.	
**ECAPM08**: Wenn die Gesundheit meines Kindes durch eine falsche oder unbedachte Entscheidung von mir Schaden nehmen würde, könnte ich mir das nie verzeihen.		If my child's health was harmed by a wrong or careless decision of mine, I could never forgive myself.	
**ECAPM09**: Es ist mir wichtig, alles zu tun, was zum Schutz der Gesundheit meines Kindes beitragen könnte.		It is important to me to do everything that might help protect my child's health	
**ECAPM10**: Für die Gesundheit meines Kindes hat das Thema Allergien einen besonders hohen Stellenwert.		For my child's health, the topic of allergies has a particularly high priority	

Original item (German version)	Response categories (German version)	Translation[1)]	Response categories
**Risc perception**			
**RP01**: Für wie wahrscheinlich halten Sie es, dass das Thema Allergien langfristig ein wichtiges Thema im Leben Ihres Kindes sein wird?	Six-point Likert scale:<br>1 = Sehr unwahrscheinlich;<br>6 = Sehr wahrscheinlich	How likely do you think it is that the topic of allergies will be an important topic in your child's life in the long term?	Six-point Likert scale:<br>1 = Very unlikely; 6 = Very likely
**RP02**: Für wie wahrscheinlich halten Sie es, dass die Lebensqualität Ihres Kindes im Laufe des Lebens durch allergische Erkrankungen deutlich beeinträchtigt wird?		How likely do you think it is that your child's quality of life will be significantly affected by allergic diseases during lifetime?	
**RP03**: Wie schätzen Sie das Risiko Ihres Kindes, langfristig unter Allergien zu leiden, im Vergleich zu dem Risiko anderer Kinder ein?	7-level scale:<br>1 = Sehr viel geringeres Risiko als bei anderen Kindern;<br>7 = Sehr viel höheres Risiko als bei anderen Kindern	How do you assess the risk of your child suffering from allergies in the long term compared to the risk of other children?	7-level scale:<br>1= much lower risk than other children;<br>7 = much higher risk than other children
**RP04**: Ich habe mir bereits Gedanken über mögliche gesundheitliche Probleme meines Kindes aufgrund von Allergien gemacht.	6-point Likert scale:<br>1 = Trifft überhaupt nicht zu;<br>6 = Trifft in sehr hohem Maße zu	I have already thought about possible health problems of my child due to allergies.	6-point Likert scale:<br>1 = Does not apply at all;<br>6 = Applies to a very high degree
**RP05**: Allergien stehen mit vielen Problemen der psychischen Gesundheit in Zusammenhang.		Allergies are associated with many mental health problems.	
**RP06**: Allergien stehen mit vielen weiteren Problemen der körperlichen Gesundheit in Zusammenhang.		Allergies are associated with many other physical health problems.	
**RP07**: Der Gedanke, dass mein Kind eine Allergie hat oder bekommen könnte, macht mir Sorgen.		Thinking that my child has or could get an allergie worries me.	
**RP08**: Für mich ist das Thema Allergien ein sehr wichtiges Thema.		Allergies are a very important topic for me.	
**RP09**: Ich mache mir Sorgen, dass die Lebensqualität meines Kindes durch Allergien langfristig beeinträchtigt wird.		I am worried that my child's quality of life will be affected by allergies in the long-term.	
**RP10**: Allergien stellen ein zunehmendes Problem für die Gesundheit in unserer Gesellschaft dar.		Allergies are an increasing health problem in our society.	
**RP11**: Wenn mein Kind Symptome einer Allergie zeigt, macht mir das Angst.		It scares me when my child shows symptoms of an allergy.	

**RP12**: Ich befürchte, dass die gesundheitliche Entwicklung meines Kindes durch Allergien erheblich beeinträchtigt wird.		I am concerned that my child's health development will be significantly affected by allergies.	
**Control belief**			
**CB01**: Wenn mein Kind Beschwerden hat, suche ich immer einen Arzt auf.	6-point Likert scale:<br>1 = Trifft überhaupt nicht zu;<br>6 = Trifft in sehr hohem Maße zu	If my child has any complaints, I always seek medical attention	6-point Likert scale:<br>1 = not true at all; 6 = very true
**CB02**: Es liegt an mir, wenn die Beschwerden meines Kindes nachlassen.		It's up to me when the complaints of my child subside.	
**CB03**: Ob die Beschwerden meines Kindes länger andauern, hängt vor allem vom Zufall ab.		It mainly depends on chance whether my child's symptoms persist for a long time	
**CB04**: Wenn ich auf mein Kind achte, bleibt es von Beschwerden verschont.		If I take care of my child, it remains free of complaints.	
**CB05**: Wenn ich genügend über mein Kind weiß, kann ich ihm selbst am besten helfen.		If I know enough about my child, I can best help them myself.	
**CB06**: Wenn ich keinen guten Arzt habe, leidet mein Kind häufiger unter Beschwerden.		If I don't have a good doctor, my child is more likely to suffer from complaints.	
**CB07**: Ob es meinem Kind gut geht oder nicht, lässt sich nicht beeinflussen.		There is no way to affect if my child is doing well or not.	
**CB08**: Ich bin der Meinung, dass Glück und Zufall eine große Rolle für das Wohlbefinden meines Kindes spielen.		I believe that luck and chance play a big role in my child's well-being.	
**CB09**: Wenn sich mein Kind unwohl fühlt, wissen andere am besten, was ihm fehlt.		When my child feels uncomfortable, others know best what is the matter.	
**Self-efficacy**			
Wenn es um die Gesundheit meines Kindes geht,…		*When considering my child´s health....*	
**SE01**: (…) weiß ich, wie ich mich verhalten soll.	6-point Likert scale:<br>1 = Trifft überhaupt nicht zu;<br>6 = trifft in sehr hohem Maße zu	(…) I know how to act.	6-point Likert scale:<br>1 = completely disagree;<br>6 = completely agree
**SE02**: (…) bin ich schnell verunsichert, weil ich etwas falsch machen könnte.		(…) I quickly become insecure because I might do something wrong.	
**SE03**: (…) gelingt es mir, die richtigen Entscheidungen zu treffen.		(…) I am able to make the right decisions.	
**SE04**: (…) fühle ich mich oft überfordert.		(…) I often feel overstrained.	
**SE05**: (…) finde ich auch dann eine Lösung, wenn Probleme auftreten.		(…) I find a solution even when problems arise.	

**SE06**: (…) verhalte ich mich so, wie ich es als Kind selbst mitbekommen habe.		(…) I behave the way I experienced my-self as a child.	
**SE07**: (…) fällt es mir leicht, so zu handeln, wie ich es für richtig halte.		(…) It is easy for me to act as I see fit.	
**SE08**: (…) kann ich auf mein Urteil und meine Fähigkeiten vertrauen.		(…) I can trust my judgment and skills.	
**SE09**: (…) muss ich zuerst wissen, wie sich andere Eltern verhalten.		(…) I first need to know how other par-ents behave.	
**SE10**: (…) mache ich immer alles richtig.		(…) I always do things right.	

1) This is a simple translation by the authors without back and forth translation. 2) Relates to a fictitious predefined recommendation for effectively reducing a child's allergy risk
